# Cystic echinococcosis involving the cardiac interventricular septum

**DOI:** 10.1590/0037-8682-0499-2020

**Published:** 2021-03-08

**Authors:** Bahar Yılmaz Çankaya, Abdurrahim Çolak

**Affiliations:** 1 Atatürk University, Department of Radiology, Erzurum, Turkey.; 2 Atatürk University, Department of Cardiovascular Surgery, Erzurum, Turkey.

**FIGURE 1:**
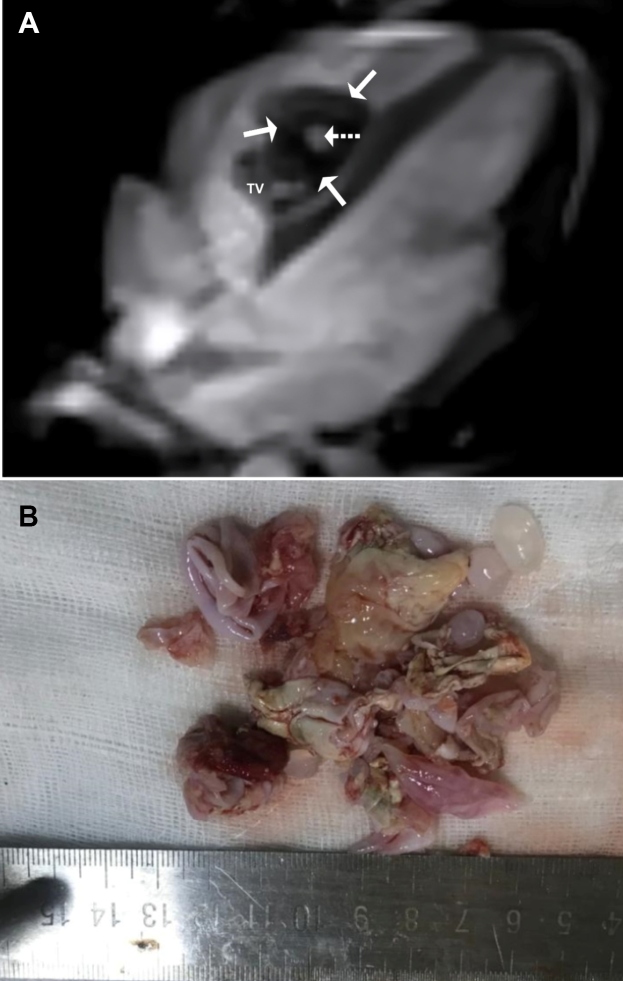
(A) Four-chamber steady-state free precession cine MR image. Hyperintense cystic areas (dashed arrow) can be seen in the hypointense mass (arrows) protruding from the interventricular septum into the right ventricle.(B) Cyst material surgically extracted from the lung and heart. TV: tricuspid valve.

A 20-year-old woman was admitted to the cardiology department with dyspnea and chest pain that commenced two months earlier. Her heart rate was 82 beats/min, respiratory rate was 24 breaths/min, and blood pressure was 120/85 mmHg. No abnormalities except eosinophilia were present in laboratory tests-white blood cell count 8200/μL (eosinophils, 15.6%), hemoglobin 12.3 g/dL, sodium 140 mmol/L, and potassium 4.3 mmol/L. Electrocardiography showed no ischemic changes. Echocardiography revealed a heterogeneous mass adhering to the septum in the right ventricle. Cardiac magnetic resonance imaging (MRI) revealed a cystic mass (43 × 35 × 28 mm) associated with the interventricular septum, protruding into the right ventricular cavity (Figure 1A). There was a cystic lesion in the right lung parenchyma. We diagnosed hydatid cyst based on the radiological characteristics, however, serological tests were negative for hydatidosis. Albendazole 400 mg was administered twice daily for five days for preoperative sterilization. We surgically removed the cysts in the right lung and heart (Figure 1B). Medical treatment was recommended for 16 weeks postoperatively, and she was discharged in good health.

Cardiac involvement due to cystic echinococcosis is rare and may manifest as angina, arrhythmia, valve dysfunction, and pericardial reaction^1^. Diagnostic tools include serological tests (indirect hemagglutination and enzyme-linked immunosorbent assay) and cardiac imaging (echocardiography, computed tomography, and MRI). However, serological tests have a 30% false-negative error rate^2^. Except in cases of multiple cysts or cardiomyopathy in which it is contraindicated, surgery is the first choice of therapy^3^.

The local ethics committee approval was obtained.
